# LOAD3: A new preclinical mouse model that incorporates humanized *APOE4*, Aβ and *MAPT*

**DOI:** 10.1002/alz70855_104914

**Published:** 2025-12-24

**Authors:** Gareth R Howell, Dylan Garceau, Kevin P Kotredes, Ravi S Pandey, Catrina Spruce, Alison Burman, Gregory W Carter, Michael Sasner

**Affiliations:** ^1^ University of Maine, Orono, ME, USA; ^2^ The Jackson Laboratory, Bar Harbor, ME, USA; ^3^ Tufts University Graduate School of Biomedical Sciences, Boston, MA, USA; ^4^ Tufts University, Boston, MA, USA; ^5^ The Jackson Laboratory for Genomic Medicine, Farmington, CT, USA

## Abstract

**Background:**

The MODEL‐AD (Model Organism Development and Evaluation for Late‐Onset Alzheimer's Disease) Consortium is creating a new generation of mouse models for Alzheimer's disease (AD) based on genetic and environmental factors shown to increase risk for human AD. The IU/JAX/PITT MODEL‐AD Center has focused particularly on strains that incorporate the variations in apolipoprotein E (*APOE*). We initially created an allelic series of *APOE* (*APOE2, APOE3, APOE4*) on the C57BL/6J (B6J) genetic background. We then created and characterized platform strains LOAD1 (*APOE4*.Trem2*R47H, double homozygous) and LOAD2 (APOE4.Trem2*R47H.hAβ, triple homozygous). Here we introduce the third strain in the series, LOAD3 (also known as LOAD3*E4) that is triple homozygous for *APOE4*, hAβ, and humanized *MAPT* (*hMAPT*), and its counterpart LOAD3*E3, created in collaboration with the BU/JAX/IU TOX‐AD center, which incorporates *APOE3* instead of *APOE4*.

**Method:**

The *APOE4* allele was created using exon replacement in B6J. *APOE3* was created through gene editing the *APOE4* allele. The humanized Aβ (hAβ) allele was created through gene editing to humanize the mouse Ab sequence. The humanized *MAPT* allele (*hMAPT*) was created through genome replacement to humanize the entire mouse *Mapt* gene (including flanking sequencing) with the H1 haplotype of the human *MAPT* gene.

**Result:**

The LOAD3 and LOAD3*E3 strains were created by crossing mice carrying either one or two of the required alleles until mice were identified carrying all three alleles. These were then further intercrossed for multiple generations to homozygose all three required alleles. All alleles are congenic on B6J. Genotypes were confirmed by PCR and sequencing and each strain is maintained as closed colonies. Both strains are viable and fertile. Cohorts of male and female LOAD3, LOAD3*E3 and B6 control mice are being created, aged and extensively phenotyped at 4, 12, 18 and 24 months of age (mos) focusing on human relevant assays. Initial phenotyping data at 4 and 12 mos will be presented and include plasma and brain biomarkers, brain multi‐omics (transcriptomics, proteomics) and neuropathology.

**Conclusion:**

LOAD3 (LOAD3*E4) and LOAD3*E3 are platform strains to enhance preclinical studies for AD. MODEL‐AD and TOX‐AD are using LOAD3 strains to evaluate additional genetic and environmental risk factors for ADRD.

